# Survived sudden cardiac death in a patient with arrhythmic mitral valve prolapse syndrome: a case report

**DOI:** 10.1007/s00392-023-02195-3

**Published:** 2023-04-12

**Authors:** Fabienne Kreimer, Andreas Mügge, Michael Gotzmann

**Affiliations:** Department of Cardiology and Rhythmology, St. Josef-Hospital, Ruhr University Bochum, Bochum, Germany

Sirs,

A 49-year-old previously healthy patient collapsed during a musical performance. Lay resuscitation was immediately started by the audience members. Return of spontaneous circulation was achieved after 16 min of defibrillator resuscitation for ventricular fibrillation.

The medical history was completely unremarkable and there was no evidence of cardiovascular disease or sudden cardiac death in the family history. The patient was a competitive athlete until a few years ago and has never experienced any symptoms such as dyspnea or palpitations before. He was not taking any medications or illicit substances.

Physical examination revealed a 4/6 noisy systolic murmur with punctum maximum over the mitral valve. The ECG demonstrated subtle biphasic T waves in the inferior leads and leads V4–V6 (Fig. 1E). Initially, there were no laboratory abnormalities, especially no cardiac troponin elevations, electrolyte disturbances, or abnormal thyroid-stimulating hormone levels. Coronary angiography did not reveal any stenoses of the coronary vessels, so that acute coronary syndrome could be excluded. CT thorax and CT angiography revealed no evidence of aortic dissection or pulmonary artery embolism. By cranial CT, intracranial hemorrhage as a cause of cardiovascular arrest has been ruled out. Cardiac MRI revealed no evidence of myocarditis or other myocardial disease. Transthoracic echocardiography demonstrated severe central mitral regurgitation in a normal sized left ventricle with good systolic pump function without regional wall motion abnormalities. Left atrial volume was slightly increased (96 mL), the left ventricular end-systolic diameter was 43 mm. Vena contracta width was 9 mm, whereas regurgitant volume was 67 mL/beat and effective regurgitant orifice area was 47 mm^2^. Furthermore, mitral annular disjunction (12 mm) was evident (Fig. 1B). Transesophageal echocardiography revealed findings of bileaflet mitral valve prolapse (MVP) with markedly thickened valve leaflets, particularly with predominance on the posterior leaflet (Fig. 1A, C).

**Fig. 1:**
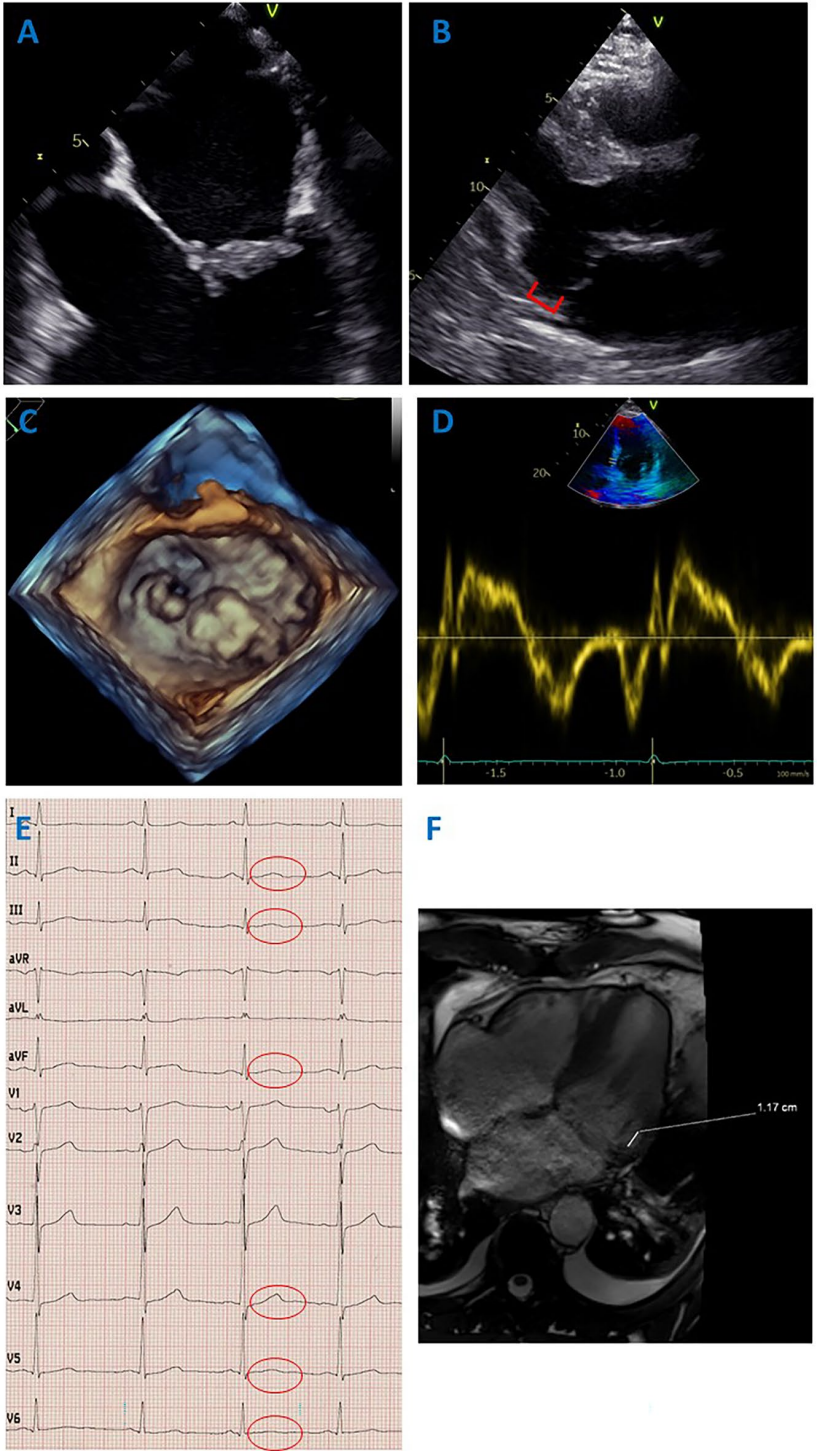
Demonstrating the clinical findings in this patient. **A** Thickening of the mitral valve leaflets, primarily with accentuation of the posterior leaflet. **B** Mitral annular disjunction on transthoracic echocardiography. Mitral annular disjunction is defined as systolic separation between the ventricular myocardium and the mitral annulus supporting the posterior mitral leaflet. **C** Bileaflet mitral valve prolapse on 3-dimensional echocardiography. **D** Tissue Doppler showing no Pickelhaube sign. **E** 12-lead ECG showing subtle biphasic T waves in the inferior leads and leads V4–V6. **F** Cardiac MRI demonstrating a mitral annular disjunction of 11.7 mm

Combining the findings and excluding other potential differential diagnoses, the diagnosis of arrhythmic MVP syndrome was made. The patient received continuous ECG monitoring during his hospital stay, in which no ventricular arrhythmias could be detected. During the hospitalization, the decision was made to implant a secondary prophylactic ICD, which was performed without complications. The patient could be discharged 10 days after surviving cardiac arrest. An initial ICD control was performed about 4 weeks later, which revealed regular ICD function and setting and no ventricular arrhythmias in the device memory. The patient denied events and symptoms. Two months after surviving sudden cardiac death, the patient underwent surgical mitral valve repair without complications.

Mitral valve prolapse (MVP) can be detected by echocardiography in 2–3% of the general population, often as an incidental finding [1]. While the vast majority of MVP patients have a good long-term outcome, depending on the extent of mitral regurgitation and its impact on the left ventricle, there also appear to be individuals at increased risk for ventricular arrhythmias and sudden cardiac death, with an estimated annual incidence of 0.2–0.4% [2, 3]. Risk stratification to decide whether a benign or malignant form of MVP is present remains challenging [4]. Herein, we report a patient with arrhythmic MVP syndrome who survived sudden cardiac death and review features associated with increased risk of ventricular arrhythmias.

Important clinical risk factors for the presence of arrhythmic MVP syndrome include unexplained syncope, palpitations, chest pain, and dyspnea [4, 5]. In general, symptomatic patients with MVP appear to be at higher risk for ventricular arrhythmias [4]. In particular, if the ventricular arrhythmias occur during exercise, the risk of sudden cardiac death is increased [6, 7]. In contrast, the patient described was completely asymptomatic as an endurance athlete both at rest and during exercise.

Family history of sudden cardiac death is relevant to weigh the possibility of inherited arrhythmia syndromes (long-QT syndrome, Brugada syndrome, arrhythmogenic right ventricular cardiomyopathy, catecholaminergic polymorphic ventricular tachycardia) as alternative diagnoses [4]. In addition, a filamin A variant has been associated with arrhythmic MVP [8]. However, routine genetic testing for non-syndromic MVP is not currently warranted [4].

Electrocardiographic features of arrhythmic MVP syndrome may include inverted or biphasic T waves, especially in the inferior and lateral leads [9]. This was also present in our patient (Fig. 1E). In addition, QT prolongations, fragmented QRS complexes, or premature ventricular contractions, have been reported in other cases of arrhythmic MVP syndrome, but were not apparent in the patient [4].

Furthermore, an increased risk of ventricular arrhythmias in the presence of a bileaflet MVP on echocardiography has been described before [4, 9]. Moreover, the risk of sudden cardiac death increases with the severity of mitral regurgitation [10]. The patient had involvement of both valve leaflets as well as severe mitral regurgitation (Fig. 1A, C). Another strong risk factor for the presence of arrhythmic MVP syndrome is mitral annular disjunction, which is characterized as systolic separation between the ventricular myocardium and the mitral annulus supporting the posterior mitral leaflet [4, 11]. A further phenomenon associated with arrhythmic MVP syndrome is mitral annulus curling, which is defined as an unusual systolic movement of the posterior mitral annulus toward the adjacent myocardium [4]. Mitral annular disjunction, but not mitral annulus curling, was also detected echocardiographically in our patient (Fig. 1B).

The Pickelhaube sign is a spiked tissue Doppler velocity profile that is also associated with arrhythmic MVP syndrome [12]. This could not be demonstrated in our patient (Fig. 1D).

In addition, cardiac MRI may be helpful in establishing the diagnosis, since an association of late gadolinium enhancement in the papillary muscle region or patchy myocardial fibrosis in the left ventricular infero-basal region with the occurrence of ventricular arrhythmias has been described [13]. However, cardiac MRI was unremarkable in this patient except for the detection of mitral annular disjunction of 11.7 mm (Fig. 1F).

Management of arrhythmic MVP syndrome is challenging because of lack of evidence from larger trials. Antiarrhythmic drugs, ablation of ventricular arrhythmias, and primary and secondary prophylactic ICD implantation may be considered [4]. In the patient who survived sudden cardiac death, secondary prophylactic ICD implantation was indicated according to current ESC guidelines [14]. In contrast, primary prophylactic ICD implantation in individuals with MVP is considered very critical because studies are lacking.

In our patient, mitral valve surgery was indicated because of the severe mitral regurgitation [15]. However, surgical intervention for arrhythmic MVP syndrome remains controversial to date. Mitral valve surgery may reduce the burden of malignant ventricular arrhythmias in MVP patients and severe mitral regurgitation, but results are inconsistent [4]. Even more critically, the use of mitral valve surgery as the sole therapeutic approach in patients at high risk for malignant ventricular arrhythmias (i.e., without ICD implantation) or its value in patients with mild to moderate mitral regurgitation remains to be questioned [4].

The arrhythmic MVP syndrome has recently come into increasing clinical and scientific focus, and efforts have been made toward a better definition to allow risk stratification in individuals with MVP (Table 1). The characteristics associated with ventricular arrhythmias and sudden cardiac death are predominantly based on single-case reports and studies with small sample sizes, resulting in a very heterogeneous entity. Pathological findings in our patient included minimal T-wave changes, bileaflet MVP with severe mitral regurgitation, and mitral annular disjunction. The etiologic workup revealed rather nonspecific findings, highlighting the challenge of risk stratification in patients with  MVP.

**Table 1:** Risk stratification in arrhythmic mitral valve prolapse syndrome

Risk stratification method	Recommended by expert consensus statement	May be recommended by expert consensus statement	Not recommended by expert consensus statement
Clinical evaluation	In patients with MVP, a careful clinical evaluation, including family history of sudden cardiac death, previous syncope, comorbidities, and physical examination, should be performed		
	Periodic clinical re-evaluation is recommended because of the possibility of disease progression or when clinical circumstances have changed (e.g., occurrence of syncope or palpitations)		
ECG monitoring	In all patients with MVP, standard 24-h Holter monitoring is warranted	Longer ECG recordings (up to 7 days) may be beneficial in selected cases to allow more accurate quantification of premature ventricular complex burden and/or to allow correlation with symptoms	
	MVP patients with complex ventricular ectopy (e.g., rapid nonsustained ventricular tachycardia) should be closely monitored	Due to the possibility of disease progression, periodic Holter may be advised as part of the routine follow-up of arrhythmic MVP patients	
		Periodic Holter monitoring of PVC burden and periodic echocardiographic evaluation of LV function may be particularly helpful in patients with frequent PVC, even when asymptomatic and with normal LV function (to allow for a timely diagnosis of premature ventricular complex induced cardiomyopathy)	
		Longer ECG recording may be advised in patients with MVP and doubtful symptoms (e.g., recurrent pre-syncope or palpitations) in whom 24 h Holter monitoring was not revealing	
		ILR may be advised in patients with MVP and unexplained syncope in whom non-invasive ECG monitoring was not revealing or inconclusive	
		ILR may be advised in patients with arrhythmic MVP, high risk features^a^ and negative CMR	
		ILR may be advised in patients with arrhythmic MVP, at least 1 phenotypic risk feature^b^ and positive LGE on CMR	
Echocardiographic protocol	In patients with suspected arrhythmic MVP, comprehensive echocardiography assessment should include evaluation of leaflet length and thickness measurement, annular dimension, MAD characterization, degenerative MR grading and possibly advanced assessment of LV function	Due to the possibility of disease progression, periodic complete transthoracic echocardiography may be advised as part of the routine follow up of arrhythmic MVP patients	
	In the work up of patients with frequent premature ventricular complexes, syncope or aborted cardiac arrest with no other obvious etiology, the comprehensive echocardiographic study should include the careful assessment of the mitral valve and the mitral annulus to diagnose arrhythmic MVP		
CMR protocol	CMR should be performed in all arrhythmic MVP patients who survived a cardiac arrest or experienced sustained ventricular arrhythmias, before a implanting an ICD for secondary prevention	CMR may be useful in patients with arrhythmic MVP and at least one phenotypic risk feature^a^	
	CMR should be performed in all patients when echocardiography does not provide accurate assessment of LV and RV function and/or evaluation of structural changes		
	CMR should be performed in all MVP patients with a history of unexplained syncope and/or non-sustained ventricular tachycardia		
	CMR should include assessment of LV size and function, assessment of MR severity, leaflet length/thickness measurement, MAD characterization and curling, and LGE assessment		
Electrophysiological study			While the committee does not endorse the use of electrophysiological studies with programmed ventricular stimulation, if electrophysiological study is used, the induction of sustained monomorphic ventricular tachycardia should be viewed as more specific than polymorphic ventricular tachycardia or ventricular fibrillation
Exercise stress testing	In patients with arrhythmic MVP, exercise stress testing should be used to assess for adrenergic-dependent rhythm disturbances	The use of exercise stress testing to assess exercise tolerance in AMVP patients who want to engage in sports is reasonable	
		Exercise stress testing (plus echocardiography), alongside other risk factors may be used to assess the clinical likelihood of obstructive coronary artery disease	

The content of this table is from reference [4]

*AMVP* arrhythmic mitral valve prolapse, *CMR* cardiac MRI, *ICD* implantable cardioverter defibrillator, *ILR* implantable loop recorder, *LGE* late gadolinium enhancement, *LV* left ventricular, *MAD* mitral annular disjunction, *MVP* mitral valve prolapse, *PVC* premature ventricular contraction

^a^High risk features: sustained ventricular tachycardia (hemodynamically tolerated), non-sustained ventricular tachycardia, unexplained syncope

^b^Phenotypic risk features: palpitations, T-wave inversion in the inferior leads, repetitive documented polymorphic premature ventricular complexes, mitral annular disjunction phenotype, redundant mitral valve leaflets, enlarged left atrium or ejection fraction ≤ 50%

## Data Availability

The data are available on request from the corresponding author.
